# Eyes and Ears: Cross-Modal Interference of Tinnitus on Visual Processing

**DOI:** 10.3389/fpsyg.2018.01779

**Published:** 2018-09-24

**Authors:** Zhicheng Li, Ruolei Gu, Xiangli Zeng, Min Qi, Jintian Cen, Shuqi Zhang, Jing Gu, Qi Chen

**Affiliations:** ^1^Department of Otolaryngology Head and Neck Surgery, The Third Affiliated Hospital, Sun Yat-sen University, Guangzhou, China; ^2^Center for Studies of Psychological Application and Department of Psychology, South China Normal University, Guangzhou, China; ^3^Key Laboratory of Behavioral Science, Institute of Psychology, Chinese Academy of Sciences, Beijing, China; ^4^Department of Psychology, University of Chinese Academy of Sciences, Beijing, China

**Keywords:** tinnitus, cross-modal interference, bottom-up, reaction time, letter symbol, emotional face

## Abstract

The visual processing capacity of tinnitus patients is worse than normal controls, indicating cross-modal interference. However, the mechanism underlying the tinnitus-modulated visual processing is largely unclear. In order to explore the influence of tinnitus on visual processing, this study used a signal recognition paradigm to observe whether the tinnitus group would display a significantly longer reaction time in processing the letter symbols (Experiment 1) and emotional faces (Experiment 2) than the control group. Signal detection and signal recognition, which reflect the perceptual and conceptual aspects of visual processing respectively, were manipulated individually in different conditions to identify the pattern of the cross-modal interference of tinnitus. The results showed that the tinnitus group required a significantly prolonged reaction time in detecting and recognizing the letter symbols and emotional faces than the control group; meanwhile, no between-group difference was detected in signal encoding. In addition, any gender- and distress-modulated effects of processing were not found, suggesting the universality of the present findings. Finally, follow-up studies would be needed to explore the neural mechanism behind the decline in speed of visual processing. The positive emotional bias in tinnitus patients also needs to be further verified and discussed.

**Highlights**:

- The bottom-up visual processing speed is decreased in tinnitus patients.

- Tinnitus primarily interferes with the detection of the visual signals in individuals.

## Introduction

In daily life, the human brain often deals with information from different sensory channels. When the brain is unable to effectively process all the information due to the limitation of cognitive resources, different sensory channels would compete with each other to fulfill the needs of information processing; this phenomenon is termed cross-modal interference (Mazza et al., 2007; Koelewijn et al., 2010).

Tinnitus is a subjective auditory experience that emerges independent of external stimuli, and its occurrence and maintenance require attention (Roberts et al., 2013). Studies have showed cross-modal interference in individuals with tinnitus, that is, visual processing in tinnitus patients is impaired compared to normal controls. For example, Stevens et al. (2007) found that the severe tinnitus group showed a significantly worse efficiency than the controls in the Stroop task, and the between-group differences increased as a function of the difficulty of the task. Araneda et al. (2015) observed similar findings in a visual-spatial Stroop task, and found out a longer reaction time (RT) and a higher error rate in the tinnitus group compared to the control group.

In what way does tinnitus modulate visual processing? According to the findings by Araneda et al. (2015), the signal detection and signal recognition tasks did not show any difference between the tinnitus and control groups. Similarly, in a visual attention network task, only the top-down executive control function of attention was affected in tinnitus group, while alerting and orienting were not significantly different from the normal group (Heeren et al., 2014). These findings indicated that tinnitus affects visual processing by interrupting the top-down visual processing with respect to executive processes, while the bottom-up stages (including signal detection and recognition) remain unchanged.

However, in the signal detection task reported by Araneda et al. (2015), the RT of the tinnitus group was longer than the control group, although the between-group difference failed to reach significance. These insignificant results might be attributed to the relatively small sample size (*n* = 17). In addition, their study investigated signal detection and recognition in independent tasks, wherein the target stimuli were different, which might have been a confounding factor. Thus, the interference of tinnitus on early visual processing awaits further investigation. We proposed that investigating signal detection and recognition in the same task would help unraveling the mechanism of the cross-modal interference of tinnitus on visual processing.

Another factor being considered in this study is the spatial characteristic of the cross-modal interference. Tinnitus symptoms are not necessarily bilateral; instead, many patients reported only one tinnitus ear (left/right). It is unknown whether the laterality of tinnitus would lead to impairment of visual processing in corresponding orientation, regarding that the allocation of attentional resources would be affected (Chica et al., 2014). To our knowledge, previous studies focusing on visual processing in tinnitus patients presented the target stimuli in the center of the screen, while the spatial factor was neglected. In contrast, the current study investigated the potential attentional bias of tinnitus patients associated with the laterality of their symptoms.

This study used letter symbols (Experiment 1) and emotional faces (Experiment 2) as the target stimuli to explore the processing of visual stimuli in tinnitus patients. Signal detection and signal recognition were disassociated by manipulating the task instructions. Specifically, in Condition 1, the subjects were asked to respond to the position (perceptual feature) of the target stimulus as soon as possible; however, they were not required to identify the content of the target. Thus, only signal detection was required in this condition. In Condition 2, the subjects were asked to judge the content (conceptual feature) of the target stimulus immediately, thus signal recognition was needed. Therefore, the RT in Condition 1 would reflect the time needed for signal detection, while Condition 2 would reflect the time needed for signal recognition. Moreover, the RT in Condition 2 subtracted from that of Condition 1 defining the time needed for signal encoding (i.e., the psychological process that translate information from sensory organs into meaningful objects). Meanwhile, this study also explored the spatial bias in visual processing of tinnitus patients by randomly presenting target stimuli on either side (left/right) of the screen.

Since tinnitus occupies an individual’s attention resources, we speculated that the tinnitus group would show a significantly lower speed to complete visual processing than the control group, indicating the effect of cross-modal interference. However, whether tinnitus would selectively modulate signal detection or signal encoding is yet to be elucidated. In addition, seeing that tinnitus might affect attentional allocation, we investigated whether the visual processing of tinnitus group would show a spatial bias; that is, the response speed of tinnitus patients to target presentation on the tinnitus side would be significantly different from that on the non-tinnitus side.

## Materials and Methods

### Participants

Patients admitted to the Outpatient Department of Otorhinolaryngology, the Third Affiliated Hospital of Sun Yat-sen University, due to tinnitus as the first complaint, were selected. The patients who fulfilled the following inclusion criteria were included in the study: (1) subjective tinnitus (non-pulsatile); (2) persistent for >6 months (chronic); (3) without hyperacusis; (4) no history of neurological and psychiatric diseases; (5) had normal vision or corrected vision; (6) an education level of high school or above and understood the operational instructions; (7) age 18–40 years; (8) right-handedness. The exclusion criteria were as follows: (1) encountered significant life events (promotion, divorce, unemployment) within 2 weeks before the experiment; (2) administered sedative or psychotropic drugs within 24 h before the experiment. Finally, a total of 38 patients (19 patients with left tinnitus and 19 patients with right tinnitus) were enrolled in the tinnitus group (15 males, 23 females, mean age = 28.87 ± 6.58 years). The present experimental protocol was reviewed and approved by the Ethics Committee of the Third Affiliated Hospital of Sun Yat-sen University. All participants signed the informed consent before the experiment.

Normal controls were recruited from the Internet and poster adverts at the Sun Yat-sen University. The inclusion criteria were as follows: (1) had no history of tinnitus, dizziness, hearing loss, and other ear diseases; (2) had no history of neurological and psychiatric diseases; (3) had normal vision or corrected vision; (4) had an education level of high school or above and could understand the operational instructions; (5) age 18–40 years; (6) right-handedness. The exclusion criteria were the same as that for the tinnitus group. Consequently, 27 participants were enrolled in the control group (9 males, 18 females, mean age = 26.70 ± 5.13 years).

Tinnitus patients were asked to complete the Tinnitus Handicap Inventory (THI) and Depression Anxiety and Stress Scale (DASS), while the controls were required to complete only the DASS. THI was used to measure the distress of tinnitus in the daily life of the patients. According to the THI grading standard issued by the British Association of Otolaryngologists, Head and Neck Surgeons in 2001 (McCombe et al., 2001), a score of ≤36 was defined as non-tinnitus distress, while a score of ≥38 was defined as tinnitus distress. Moreover, DASS indicated the levels of depression, anxiety, and stress in subjects (**Table 1**).

**Table 1:** Comparison of age and DASS assessment between the groups.

	Tinnitus group (*n* = 38)	Control group (*n* = 27)	*P*
Age	28.87 ± 6.58	26.70 ± 5.13	0.16
Depression	5.21 ± 5.96	4.56 ± 3.53	0.58
Anxiety	7.24 ± 5.66	5.78 ± 4.74	0.28
Stress	10.34 ± 8.11	9.78 ± 6.00	0.76


### Stimulus

**FIGURE 1:**
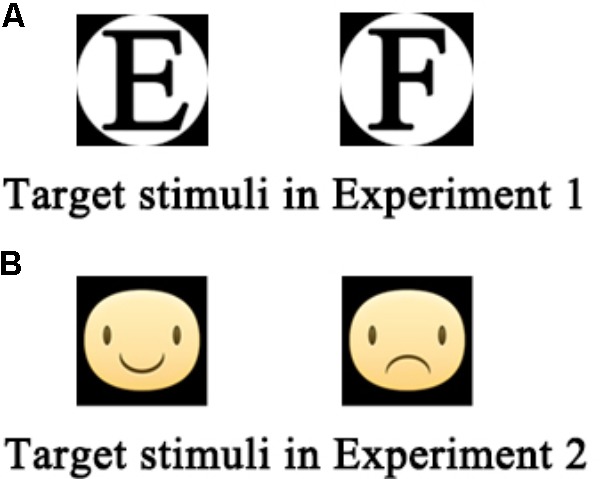
**(A,B)** Target stimuli used in Experiments 1 and 2.

In Experiment 1, two composite figures (consisting of the black letter E or F inside white circles, **Figure 1A**) were used as target stimuli.

In Experiment 2, two facial expressions were used as the target stimuli: happiness and sadness, wherein the difference was the direction of the mouth (upward vs. downward; **Figure 1B**). Prior to the experiment, 39 normal volunteers (aged 20–40 years) were recruited to assess the valence (from 1: very negative to 7: very positive) and arousal (from 1: very low to 7: very high) of the two facial expressions using two 7-point scales. The results showed that the valence and arousal ratings of the happy face were 5.23 ± 0.74 and 2.46 ± 1.33, respectively, while those of the sad face were 3.15 ± 0.74 and 2.92 ± 1.06, respectively. Paired sample *t*-tests demonstrated that the emotional valence of the happy face was significantly higher than that of the sad face (*t* = -12.34, *P* < 0.01), while the arousal did not show any significant difference between the two (*t* = -1.69, *P* = 0.10).

All stimuli were designed using Photoshop CS6 (Adobe Systems Inc., San Jose, CA, United States), with a pixel size of 100 × 100 and were displayed on a computer screen.

### Procedure

**FIGURE 2:**
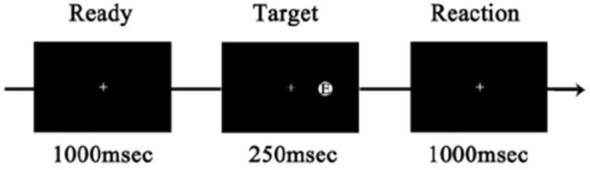
An example of the trial.

The target stimuli were displayed and the subjects’ responses recorded using Presentation 17.0 (Neurobehavioral Systems Inc., Berkeley, CA, United States). At the beginning of each trial, a white fixation point (“+”) in the center of the black screen (800 × 600) was displayed for 1,000 ms. Subsequently, a target stimulus was displayed for 250 ms at either side of the screen. The subjects pressed the left or right “Alt” key on the keyboard within 1,000 ms. The current trial would finish immediately after the subjects made a selection or 1,000 ms had passed (**Figure 2**). A total of 40 trials were conducted. The target type (E/F or Happiness/Sad) and position (left/right) were randomized and counterbalanced across trials.

In both Experiment 1 and 2, each subject was required to complete the two conditions of tasks (Conditions 1 and 2) in two independent blocks, at an interval of 10 min. In Condition 1, the subjects responded to the position of the target stimulus. (For example, if the target stimulus appeared at the right side of the screen, the subjects should press the right “Alt” key.) In Condition 2, the subjects responded to the content of the target stimulus. (For example, if the target stimulus was letter “E,” the subjects should press the left “Alt” key.)

The order of the two conditions in the whole sample was balanced between the subjects, and approximately 15 min were required to complete the entire experimental procedure.

### Data Measurements and Analysis

Data analyses were performed using SPSS 19.0 software (IBM Corporation, Armonk, NY, United States). Omissions, incorrect responses, trials with RTs three standard deviations (SDs) away from the mean RT were excluded from further analysis. Then, the mean RTs of the remaining trials were calculated. Normal distributed data were reported with mean and standard deviation. Inter-group difference and intra-group difference were evaluated by independent sample *t*-test and paired sample *t*-test, respectively. Otherwise, median and quartile range were presented, and difference was tested by Mann–Whitney Test or Wilcoxon Signed Ranks Test (normal approximation test results were reported). *P* < 0.05 was considered statistically significant.

## Results

### Proportion of Abnormal Data in Each Group

Omissions, incorrect responses, and trials with RTs that were 3 SDs away from the mean were defined as abnormal data and excluded from further analysis. The proportion of the abnormal data in each group were shown in **Table 2**.

**Table 2:** Proportion of abnormal data in each group.

	Tinnitus group	Normal group	χ^2^	*P*
**Experiment 1**				
Condition 1	1.12%	0.93%	0.23	0.63
Condition 2	1.71%	1.57%	0.07	0.79
**Experiment 2**				
Condition 1	1.25%	1.02%	0.30	0.59
Condition 2	2.11%	2.04%	0.01	0.90


### Experiment 1: Differences in Letter Symbols Recognition

The independent samples rank-test showed that the tinnitus group was significantly slower than the control group in detecting and recognizing the target stimuli, while no significant differences were observed in encoding the target stimuli (the recognition speed minus the detection speed) between the two groups (**Table 3**).

**Table 3:** Inter-group differences in letter symbols recognition.

Stage	Side	Tinnitus group	Normal group	*Z*	*P*
Detection	Left	391.67 (359.77, 456.15)	344.82 (290.45, 403.56)	-3.02	<0.01^∗^
	Right	393.88 (367.57, 432.00)	358.16 (289.84, 386.46)	-3.21	<0.01^∗^
Encoding	Left	261.95 (211.38, 299.42)	254.02 (194.02, 313.43)	-0.32	0.75
	Right	276.92 (205.41, 308.68)	256.41 (210.98, 315.42)	-0.71	0.48
Recognition	Left	668.97 (617.77, 721.19)	617.74 (580.32, 663.19)	-2.75	<0.01^∗^
	Right	664.73 (618.98, 740.62)	609.29 (556.47, 660.46)	-3.13	<0.01^∗^


*The asterisk indicates a significant statistical difference (*P* < 0.05).*

Meanwhile, paired sample *t*-test or rank-test showed that significantly lateral dominances were not observed in the left tinnitus group, right tinnitus group and the normal group in detecting, encoding and recognizing the target stimuli (**Table 4**).

**Table 4:** Lateral dominances in letter symbols recognition in each group.

Stage	Group	Left side	Right side	*t/z*	*P*
Detection	Left tinnitus	387.99 (346.44, 448.13)	396.04 (367.29, 425.08)	-0.77	0.45
	Right tinnitus	391.75 (374.69, 471.04)	391.37 (370.63, 436.39)	-0.89	0.38
	Normal	353.14 ± 70.96	348.94 ± 64.81	-0.76	0.45
Encoding	Left tinnitus	258.18 (209.95, 285.88)	275.23 (230.96, 297.37)	-1.65	0.10
	Right tinnitus	271.15 (217.83, 314.97)	278.61 (191.22, 332.69)	-0.48	0.63
	Normal	253.59 ± 64.85	257.88 ± 66.86	0.54	0.59
Recognition	Left tinnitus	652.49 (586.63, 721.04)	659.79 (617.75, 715.03)	-1.45	0.15
	Right tinnitus	673.94 (633.16, 721.65)	675.64 (647.79, 745.67)	-0.64	0.52
	Normal	606.73 ± 74.63	606.83 ± 78.99	0.01	0.99


Finally, the independent samples rank-test showed that neither gender nor tinnitus distress affected the speed in detecting, encoding and recognizing the target stimuli (**Tables 5, 6**).

**Table 5:** Effect of gender on letter symbols recognition in tinnitus patients.

Stage	Side	Male	Female	*z*	*P*
Detection	Left	391.58 (337.62, 471.04)	392.65 (374.69, 448.13)	-0.49	0.64
	Right	396.04 (350.61, 436.39)	391.72 (370.63, 425.08)	-0.11	0.93
Encoding	Left	222.55 (207.25, 287.42)	274.32 (223.63, 299.93)	-1.36	0.18
	Right	230.96 (182.92, 305.31)	292.13 (256.41, 318.78)	-1.72	0.09
Recognition	Left	650.57 (596.83, 721.65)	673.60 (625.25, 721.04)	-0.94	0.36
	Right	660.78 (598.25, 738.94)	668.68 (647.33, 745.67)	-0.49	0.64


**Table 6:** Effect of tinnitus distress on letter symbols recognition.

Stage	Side	Tinnitus distress	Non-tinnitus distress	*z*	*P*
Detection	Left	392.20 (366.03, 460,74)	389.78 (331.70, 454.54)	-0.63	0.55
	Right	400.38 (368.97, 429.17)	374.82 (318.46, 455.11)	-0.96	0.35
Encoding	Left	272.64 (210.43, 296.71)	247.47 (212.11, 300.99)	-0.23	0.83
	Right	279.00 (211.98, 325.12)	264.76 (193.65, 302.75)	-0.60	0.67
Recognition	Left	668.97 (621.67, 729.45)	673.04 (594.73, 703.87)	-0.37	0.73
	Right	666.83 (626.37, 746.86)	662.30 (592.31, 721.01)	-0.53	0.61


### Experiment 2: Differences in Emotional Face Recognition

The independent samples rank-test showed that the tinnitus group was significantly slower than the control group in detecting and recognizing the target stimuli, while no significant differences were observed in encoding the target stimuli (the recognition speed minus the detection speed) between the two groups, regardless of whether the face was happy or sad (**Table 7**).

**Table 7:** Inter-group differences in emotional face recognition.

Stage	Side	Tinnitus group	Normal group	*z*	*P*
Detection	Left	391.58 (356.54, 458.45)	341.35 (289.83, 391.64)	-3.04	<0.01^*^
	Right	391.72 (367.48, 427.81)	354.10 (289.27, 376.42)	-3.20	<0.01^*^
**Happy face**					
Encoding	Left	253.09 (202.22, 333.25)	252.73 (200.80, 299.98)	-0.28	0.78
	Right	290.22 (212.64, 367.37)	274.39 (235.61, 318.66)	-1.10	0.27
Recognition	Left	694.26 (617.52, 779.73)	624.78 (547.23, 659.04)	-2.54	0.01^*^
	Right	693.43 (637.70, 772.21)	621.48 (587.56, 670.88)	-3.34	<0.01^*^
**Sad face**					
Encoding	Left	277.15 (230.95, 371.92)	263.86 (221.93, 313.43)	-1.01	0.32
	Right	293.66 (243.06, 361.08)	263.09 (215.17, 344.45)	-1.17	0.24
Recognition	Left	696.86 (636.17, 797.58)	627.48 (553.00, 691.03)	-3.21	<0.01^*^
	Right	678.69 (632.33, 796.52)	635.84 (548.08, 686.71)	-2.88	<0.01^*^


*The asterisk indicates a significant statistical difference (*P* < 0.05).*

Meanwhile, paired sample *t*-test or rank-test showed that there was no significant lateral effect in the left tinnitus group, right tinnitus group, or normal group in detecting, encoding, and recognizing the target stimuli, regardless of whether the face was happy or sad (**Table 8**).

**Table 8:** Lateral dominances in emotional face recognition in each group.

Stage	Group	Left side	Right side	*t/z*	*P*
Detection	Left tinnitus	383.22 (344.32, 445.74)	393.88 (362.93, 415.34)	0.63	0.53
	Right tinnitus	391.75 (374.69, 471.04)	391.37 (370.63, 436.39)	-0.89	0.38
	Normal	351.20 ± 71.63	347.17 ± 65.42	-0.73	0.49
**Happy face**					
Encoding	Left tinnitus	258.86 (185.50, 339.48)	300.92 (163.74, 396.60)	1.20	0.23
	Right tinnitus	253.09 (209.34, 333.61)	284.28 (262.71, 337.51)	0.85	0.40
	Normal	271.85 ± 84.17	267.42 ± 94.59	-0.35	0.73
Recognition	Left tinnitus	684.81 (573.84, 790.24)	734.16 (596.17, 787.12)	1.07	0.29
	Right tinnitus	703.83 (619.45, 756.26)	682.37 (647.10, 730.91)	-0.68	0.49
	Normal	618.63 ± 95.01	619.02 ± 70.02	0.03	0.98
**Sad face**					
Encoding	Left tinnitus	265.50 (203.70, 297.61)	297.80 (225.97, 367.70)	0.72	0.47
	Right tinnitus	294.16 (246.36, 374.31)	279.46 (244.14, 361.50)	-0.72	0.47
	Normal	261.66 ± 85.21	272.33 ± 89.69	1.26	0.22
Recognition	Left tinnitus	673.11 (606.68, 806.79)	676.06 (604.62, 803.47)	0.68	0.50
	Right tinnitus	711.39 (652.31, 797.33)	685.50 (648.59, 797.05)	-0.64	0.52
	Normal	612.86 ± 83.81	619.50 ± 88.27	0.64	0.53


Finally, the independent samples rank-test showed that neither gender nor tinnitus distress affected the speed in detecting, encoding, or recognizing target stimuli, regardless of whether the face was happy or sad (**Tables 9, 10**).

**Table 9:** Effect of gender on emotional face recognition in tinnitus patients.

Stage	Side	Male	Female	*z*	*P*
Detection	Left	391.58 (337.62, 471.04)	390.32 (371.77, 445.74)	-0.43	0.68
	Right	396.04 (350.61, 436.39)	391.55 (369.89, 421.55)	-0.03	0.99
**Happy face**					
Encoding	Left	264.63 (209.34, 356.65)	250.28 (192.77, 332.97)	-0.46	0.66
	Right	290.22 (204.04, 373.71)	292.12 (213.02, 351.36)	-0.03	0.99
Recognition	Left	703.83 (615.59, 756.26)	663.72 (609.36, 791.59)	-0.16	0.89
	Right	696.93 (640.83, 765.39)	673.04 (632.76, 782.03)	-0.31	0.77
**Sad face**					
Encoding	Left	295.17 (256.65, 416.83)	266.18 (222.89, 301.78)	-1.49	0.14
	Right	279.46 (235.11, 361.50)	297.69 (253.35, 359.10)	-0.50	0.64
Recognition	Left	725.94 (657.99, 797.84)	678.07 (615.78, 773.39)	-0.96	0.35
	Right	686.19 (640.64, 795.99)	672.89 (617.21, 817.58)	-0.31	0.77


**Table 10:** Effect of tinnitus distress on emotional face recognition.

Stage	Side	Tinnitus distress	Non-tinnitus distress	*z*	*P*
Detection	Left	391.75 (363.15, 463.04)	389.78 (331.70, 454.54)	-0.55	0.60
	Right	397.89 (368.42, 425.08)	374.82 (318.46, 455.11)	-0.89	0.39
**Happy face**					
Encoding	Left	253.09 (216.09, 332.88)	263.48 (176.71, 360.20)	-0.51	0.63
	Right	289.29 (221.24, 361.03)	295.21 (174.38, 399.15)	-0.31	0.78
Recognition	Left	686.06 (619.45, 786.73)	699.94 (567.29, 776.96)	-0.34	0.75
	Right	688.11 (634.58, 785.82)	721.66 (621.37, 768.80)	-0.17	0.88
**Sad face**					
Encoding	Left	272.97 (234.95, 370.65)	277.42 (208.82, 387.44)	-0.17	0.88
	Right	301.73 (248.61, 361.50)	286.56 (195.55, 342.49)	-0.72	0.49
Recognition	Left	683.54 (621.05, 797.84)	719.60 (627.54, 783.91)	-0.07	0.96
	Right	676.01 (634.59, 814.81)	703.31 (602.59, 740.75)	-0.21	0.85


### Difference in Processing Between Emotional Faces

The paired sample *t*-test revealed that the difference between the RTs of happy and sad faces in the control group was insignificant, while the RTs of the happy face was significantly higher than that of the sad face in the tinnitus group in the left side, but not the right side (**Table 11**).

**Table 11:** Difference in processing between emotional faces.

Stage	Group/Side	Happy face	Sad face	*t/z*	*P*
Encoding	Tinnitus/Left	253.09 (202.22, 333.25)	277.15 (230.95, 371.92)	-2.01	0.04^*^
	Tinnitus/Right	290.22 (212.64, 367.37)	293.66 (243.06, 361.08)	-0.17	0.86
	Normal/Left	271.85 ± 84.17	261.66 ± 85.21	1.10	0.28
	Normal/Right	267.42 ± 94.59	272.33 ± 89.69	-0.50	0.62
Recognition	Tinnitus/Left	694.26 (617.52, 779.73)	696.86 (636.17, 797.58)	-2.01	0.04^*^
	Tinnitus/Right	693.43 (637.70, 772.21)	678.69 (632.33, 796.52)	-0.17	0.86
	Normal/Left	618.63 ± 95.01	612.86 ± 83.81	-0.04	0.97
	Normal/Right	619.02 ± 70.01	619.50 ± 88.27	0.53	0.60


*The asterisk indicates a significant statistical difference (*P* < 0.05).*

## Discussion

In this study, two behavioral experiments were conducted to explore the cross-modal inference of tinnitus on visual processing. The preliminary results of this study indicated that the signal detection and signal recognition were significantly declined in the tinnitus patients, irrespective of the stimulus type, which supports the first hypothesis of this study. Meanwhile, an insignificant difference was noted in the encoding speed of the target stimuli between the two groups; thus, the decrease in signal detection might be a vital factor causing the decrease in signal recognition in tinnitus patients. Finally, the lack of significant difference in the influence of gender and tinnitus distress on both types of visual processing (including detection, encoding, and recognition) indicated that the decrease in the visual processing capacity is prevalent in the chronic tinnitus population.

Meanwhile, the results of this study showed that there was no significant lateral effect in visual processing in either the tinnitus group or the normal group, and therefore can not support the second hypothesis that tinnitus might affect spatial attentional allocation in visual processing. In a previous research based on cue-target paradigm, there had the interstimulus interval (ISI) between the cue and the target (Chica et al., 2014), attention resources can be detached from cues to target stimuli, which affected the processing of target stimuli by individuals. However, the attention resources occupied by tinnitus were difficult to separate from the tinnitus signal (Li et al., 2016), thus tinnitus was hard to relate to target stimulation and cannot act as the spatial cue in visual processing.

### Cross-Modal Interference of Tinnitus on Visual Processing

Consistent with our expectation, the present study provided preliminary behavioral evidence for the cross-modal interference of tinnitus on visual processing. Specifically, the visual detection and recognition speeds of the tinnitus group to letter symbols and emotional faces were significantly slower than that of the control group, indicating that the effect of tinnitus may occur at both the perceptual and conceptual level in visual processing. Therefore, the tinnitus signal might affect the allocation of attention resources in patients, thereby interfering with the processing in the visual channel. Concurrently, the findings also revealed that the decline in the visual processing speed in tinnitus subjects was primarily due to the decline in the detection speed of the target stimuli. This phenomenon suggested the presence of the cross-modal interference of tinnitus in the early stage of visual cognitive processing.

Previous studies found that both visual and auditory spatial tasks activate the same brain area at the early stage of cognitive processing (<600 ms), which indicated that these sensory channels share the same attention regulation system at this stage (supramodal). Moreover, in the late stage of cognitive processing (600–800 ms), spatial tasks based on different channels activate different brain areas, which indicate that the visual and auditory channels have their independent attention regulation systems at this stage (sensory-specific) (Banerjee et al., 2011).

In this study, the target stimuli are randomly displayed at the two sides of the screen, as the visual spatial task. Thus, we initially speculated that the decrease in the detection speed of the tinnitus subjects to the target stimuli could be attributed to the abnormal auditory signals (tinnitus) occupying the attention resources in the supramodal, thereby weakening the ability to detect the visual signals. In addition, at the late stage of cognitive processing (encoding the target stimuli), the sensory specificity effectively alleviates the interference of the abnormal processing in the auditory channel (tinnitus) to the visual signal processing.

### Positive Emotional Advantage in Tinnitus Patients?

The “negativity bias” has long been established in the literature, i.e., negative emotions have advantages in attracting attentional resources as compared to positive emotions, and thus, individuals react quickly to negative emotions (Yiend, 2010). However, the present study revealed that the control group did not exhibit any significant difference in processing the speed between happy and sad faces. This phenomenon might be attributed to the use of abstract rather than real faces, which showed low levels of arousal. Consequently, the difference in body reaction to these two faces was insignificant (Droit-Volet and Berthon, 2017). In addition, the negative emotional pictures used in previous studies contain threatening information, such as the appearance of spiders, snakes, or angry faces, resulting in negativity bias by eliciting defensive reactions (Lobue and DeLoache, 2008; LoBue, 2009).

Meanwhile, a difference was noted in the tinnitus group, such that the RTs to happy faces were significantly shorter than that for sad faces in the left side. This behavioral pattern was in contrast with the classic negativity bias. Tinnitus was an unusual auditory experience, to which most patients felt puzzled, doubtful, and anxious (Zeng et al., 2010). These adverse reactions further enhanced the patients’ negative experience to tinnitus, which resulted in a vicious circle of negative experience and adverse reaction (Jastreboff, 1990; Li et al., 2015). In order to maintain their psychological balance and mental health, we suggest that tinnitus patients may have a general tendency to avoid the processing of negative emotions. However, the potential interferences of task design and individual difference were still largely unclear. Therefore, the findings of this study still need to be further verified in follow-up research.

### Summary and Prospects

The current study provided a preliminary behavioral evidence for the cross-modal interference of tinnitus to visual processing and suggested that the interference exists in early visual processing. However, the findings in this study required further verification.

First, the stimuli used in the research on the classic visual-auditory interference are meaningful (such as speech and orientations), while the tinnitus is a monotonous meaningless auditory experience. Thus, the mechanism for cross-modal interference of tinnitus to the visual processing may require exploration using experiments, rather than referring to the findings in classic cross-modal studies.

Second, in this study, the difference in the RTs between the tinnitus group and control group was used to measure the influence of tinnitus on signal recognition. However, it would not be surprising if the cognitive mechanisms underlying the current task are actually more complicated than our presumption. Future studies using neuroscience techniques (such as brain-imaging) may help clarify this issue.

## Conclusion

The RT of visual processing was significantly decreased in tinnitus patients, especially the signal detection speed. Further studies would be needed to explore the neural mechanism behind the decline in signal processing speed.

## Author Contributions

ZL designed and performed the experiments, analyzed the data, and wrote the paper. RG analyzed the data and perfected the paper. XZ modified research approach and chose the patients. QC directed and modified research approach and provided critical revision. ZL, RG, XZ, and QC discussed the results and implications and commented on the manuscript at all stages. MQ, JC, SZ, and JG in charge of preliminary screening and contacted with subject.

## Conflict of Interest Statement

The authors declare that the research was conducted in the absence of any commercial or financial relationships that could be construed as a potential conflict of interest.The handling Editor declared a shared affiliation, though no other collaboration, with one of the authors RG.
